# COVID-19 challenging cell biology

**DOI:** 10.1007/s00709-020-01506-z

**Published:** 2020-04-24

**Authors:** Joern Bullerdiek

**Affiliations:** 1 Institute of Medical Genetics, University Rostock Medical Center, Ernst-Heydemann-Str. 8, 18057 Rostock, Germany; 2 Human Genetics, University of Bremen, 28359 Bremen, Germany

## COVID-19 challenging cell biologists

Though its true origin is not clear at all, the sentence “May you live in interesting times!” is often quoted as a common Chinese curse. Let alone the question of whether or not this curse is indeed of Chinese origin, there is no doubt that we live in interesting times requiring efforts of cell biologists as well. Severe acute respiratory syndrome coronavirus 2 (SARS-CoV-2, syn.: 2019-nCoV) has caused the pandemic disease COVID-19. Central questions regarding the current infections deal with virus-host cell interactions, and besides vaccination the immediate interaction between these both partners is of high therapeutic relevance.

To speed up the development of therapies, research into this interaction is urgently required. One critical point is virus entry into the host cell. Quite recently, Hoffmann and coworkers have described two major human cellular tools which are used by SARS-CoV-2 to enter its host ´s cells: Attachment to the cell is mediated by the receptor Angiotensin-Converting Enzyme 2 (ACE2) followed by priming of the viral spike protein by cellular Transmembrane Serine Protease 2 (TMPRSS2) (Hoffmann et al. 2020). The latter step can be blocked by existing protease inhibitors (Hoffmann et al. 2020), while in the absence of ACE2 virus infectivity is impaired (Zhou et al. 2020). Interestingly, both tools are also used by the coronavirus causing severe acute respiratory syndrome of the 2002/2003 pandemic (SARS-CoV). This pioneering work raises a couple of further questions. Do variants of genes encoding either of these proteins contribute to the variability seen in the clinical symptoms in particular the severity and course of the disease? Does the regulation of their genes play a role? Interestingly, somewhat similar findings have recently been described for MERS-CoV engaging human Transmembrane Protein Dipeptidyl Peptidase 4 (DPP4). Some polymorphisms of *DPP4* apparently reduce the binding efficiency of MERS-CoV spike glucoprotein to DPP4 (Kleine-Weber et al., 2020).

**Fig. 1:**
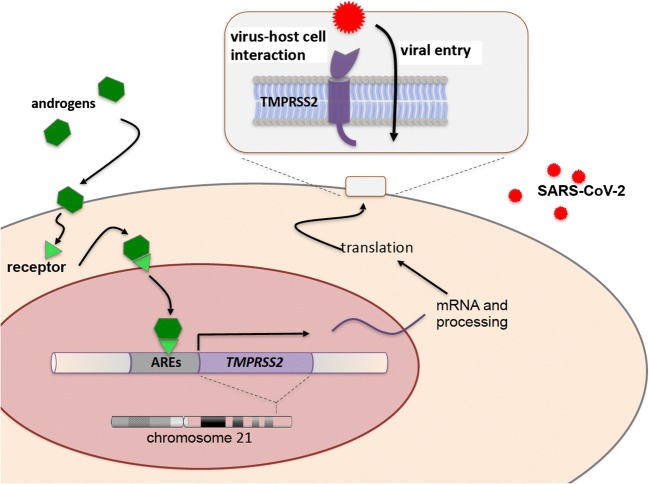
The Transmembrane Serine Protease 2 (TMPRSS2) as a tool used by SARS-CoV-2 to enter human cells. The gene (*TMPRSS2*) encoding the protease has been assigned to human chromosome 21 at q22.3. Androgen Response Elements (AREs) within its regulatory sequences are part of the positive regulation by androgens. The gene product is a transmembrane protein priming viral spike protein thereby facilitating viral entry

*TMPRSS2* is a gene well-known to tumor geneticists. Its regulatory sequences including androgen response elements drive tumorigenesis in a large percentage of prostate cancers when translocated to genes encoding certain transcription factors (Tomlins et al. 2005). Now, TMPRSS2 re-enters the stage as a player mediating viral entry. Of note, in a genome-wide association study, polymorphisms in an upstream regulatory region of *TMPRSS2* leading to its enhanced expression turned out to be associated with a higher risk for severe H1N1 influenza (Tomlins et al. 2005). If ethnicity may emerge as a risk factor for severe SARS-CoV-2 infection, polymorphisms of and around *TMPRSS2* and *ACE2* may in part explain these findings. A known risk factor, however, is male sex. Of note, in adults, *TMPRSS2* is strongly expressed in lung airway epithelium but also in the prostate and its expression is positively regulated by androgens (Fig. 1). Accordingly, canonical as well as non-canonical Androgen Response Elements (AREs) have been found in cis-regulatory sequences of *TMPRSS2* (Wang et al. 2007; Clinckemalie et al. 2013). One might therefore speculate whether this type of regulation contributes to the well-documented increased risk for males (Chen et al. 2020) that also has been reported for SARS-CoV in humans and mice (Channappanavar et al. 2017). Moreover, a single nucleotide polymorphism (rs8134378) within one of these androgen response elements reduces binding and activation by the androgen receptor (Clinckemalie et al. 2013).

Like these, many other questions regarding virus-host cell interactions wait to be tackled and the editors of *Protoplasma* are excited to receive manuscripts dealing with these aspects.
